# Impact of mobile health on maternal and child health service utilization and continuum of care in Northern Ghana

**DOI:** 10.1038/s41598-023-29683-w

**Published:** 2023-02-21

**Authors:** Abdul Ganiyu Kantamah Nuhu, Duah Dwomoh, Susan Ama Amuasi, Winfred Dotse-Gborgbortsi, Chrysantus Kubio, Edward Anane Apraku, Jonas Kolong Timbire, Justice Nonvignon

**Affiliations:** 1 Busunu Health Center, West Gonja Municipal Health Directorate, Damongo, Ghana; 2 Department of Biostatistics, School of Public Health, University of Ghana, Accra, Ghana; 3 Department of Physician Assistantship and Public Health, School of Medicine and Health Sciences, Central University College, Accra, Ghana; 4 WorldPop, School of Geography and Environmental Science, University of Southampton, Southampton, UK; 5 Savannah Regional Health Directorate, Ghana Health Service, Damongo, Ghana; 6 Kintampo Health Research Centre, Kintampo, Bono East Region, Ghana; 7 Nabdam District Health Directorate, Ghana Health Service, Nangodi, Upper East Region, Ghana; 8 Department of Health Policy, Planning and Management, School of Public Health, University of Ghana, Accra, Ghana

**Keywords:** Health care, Medical research

## Abstract

Maternal and child mortality are of public health concern. Most of these deaths occur in rural communities of developing countries. Technology for maternal and child health (T4MCH) is an intervention introduced to increase Maternal and Child Health (MCH) services utilization and continuum of care in some health facilities across Ghana. The objective of this study is to assess the impact of T4MCH intervention on MCH services utilization and continuum of care in the Sawla-Tuna-Kalba District in the Savannah Region of Ghana. This is a quasi-experimental study with a retrospective review of records of MCH services of women who attended antenatal services in some selected health centers in the Bole (comparison district) and Sawla-Tuna-Kalba (intervention district) of the Savannah region, Ghana. A total of 469 records were reviewed, 263 in Bole and 206 in Sawla-Tuna-Kalba. A multivariable modified Poisson and logistic regression models with augmented inverse-probability weighted regression adjustment based on propensity scores were used to quantify the impact of the intervention on service utilization and continuum of care. The implementation of T4MCH intervention increased antenatal care attendance, facility delivery, postnatal care and continuum of care by 18 percentage points (ppts) [95% CI - 17.0, 52.0], 14 ppts [95% CI 6.0%, 21.0%], 27 ppts [95% CI 15.0, 26.0] and 15.0 ppts [95% CI 8.0, 23.0] respectively compared to the control districts. The study showed that T4MCH intervention improved antenatal care, skilled delivery, postnatal services utilization, and continuum of care in health facilities in the intervention district. The intervention is recommended for a scale-up in other rural areas of Northern Ghana and the West-African sub-region.

## Introduction

Maternal and child mortality is a threat to public health. The World Health Organization (WHO) estimated 830 maternal deaths each day in 2015. The majority (99%) of these deaths occur in rural communities of developing countries with over half of the deaths occurring in sub-Saharan Africa. The ratio of maternal deaths in developing countries such as Ghana is 239 per 100,000 live births in 2015^1^. The WHO further estimated that 5.4 million children below 5 years died in 2017, and 2.5 million of them died within the first 28 days of life with children in sub-Saharan Africa 15 times more likely to die before the age of five compared to children in developed countries^1^.

The sixth Ghana Demographic and Health Survey (GDHS) also indicated that 97% of women who delivered 5 years preceding the survey had at least one antenatal care (ANC). Nine out of every ten women (87%) had four visits before delivery with the proportion of women attending and delivering with a skilled provider increasing from 40/1000 live births in 1998 to 74 /1000 live births in 2014^2^. Delays in identifying danger and making decisions to seek health, reaching out to appropriate caregiver as well as obtaining adequate and the right treatment is the key factor contributing to the delay in the elimination of maternal and infant mortality across our communities^3^.

The death of women during pregnancy and birth as well as deaths of children under the age of 5 years is a key concern to every nation. Maternal and Child health services utilization are known to be key initiatives in addressing this canker. According to WHO, in 2015 almost all pregnant women in developed countries had a minimum of four ANC visits and were attended to by skilled health staff during childbirth as well as postnatal care compared to 40% of those in developing countries^1^.

In the northern region of Ghana, 92% of pregnant women received an average of four ANC visits with only 35.4% delivering with a skilled attendant and as high as 78.2% of nursing mothers and their babies not receiving postnatal care (PNC) within the first 2 days of birth^4^. In the Sawla-Tuna-Kalba district, the 2018 District Health Information Management System (DHIMS2) reported that only 56.7% of pregnant women made an average of four ANC visits and as few as 38.8% of pregnant women delivered with a skilled attendant^5^.

Mobile technology penetration in health across the world has assisted in improving access to health services and reducing delays in accessing health by serving as a medium through which information on MCH services is delivered to mothers. Savana Signatures, a Non-Governmental Organization (NGO) in collaboration with Salasan. Inc. and Mustimuhw Information Solutions, with support from Global Affairs Canada, implemented Technology for Maternal and Child Health (T4MCH) project in the Sawla-Tuna-Kalba district of the Savannah Region. The project aims to increase MCH services utilization of health facilities via delivering weekly short message service (SMS) in English or voice messages in local languages within the intervention areas. Health care providers within the implementation areas were trained on Information Communication and Technology (ICT) and given smartphones and other ICT equipment to assist in collecting clients’ information. This was fed into a platform known as “Kpododo” which then generates weekly voice messages/SMS to educate and remind pregnant women and nursing mothers on MCH services with respect to their gestation via mobile network operators across Ghana^6^.

Despite significant investments in various interventions such as T4MCH by governments and other organizations across the district to improve MCH service utilization, little is known about the efficiency, cost-effectiveness, and impact of such interventions. Before the implementation of T4MCH we have had other programs Millennium Accelerated Fund program (MAF), Highly Rapid Improvement on service Delivery (HIRD), and currently, Maternal Child Health and Nutrition Program (MCHNP) being implemented across all facilities in these two districts (intervention and control) to improve on MCH services utilization.

This study assessed the impact of T4MCH project intervention on MCH service utilization in the Sawla-Tuna-Kalba district. We hypothesized that women receiving the T4MCH intervention will have a higher number of ANC attendance, a higher percentage of facility delivery, and a higher number of postnatal visits compared to non-beneficiaries.

## Methods

This study followed the standard guidelines for reporting quasi-experimental studies using the Transparent Reporting of Evaluations with Nonrandomized Design/Quasi-Experimental Study Design (TREND).

We confirm that all methods were carried out in accordance with relevant guidelines and regulations and in accordance with the declaration of Helsinki.

### Study type

A quasi-experimental study with retrospective records review was conducted at some selected health facilities in the Savanah Region between January 2019 and May 2019. A data extraction tool was used to extract data from antenatal, delivery, and postnatal care registers in the Sawla-Tuna-Kalba (intervention site) and Bole (comparison) districts, all in the Savannah Region.

### Study setting

The study was conducted at Kalba, Sawla, Tuna, and Gindabo Health Centres in the Sawla-Tuna-Kalba district as the study sites and Mankuma, Bole, Bamboi, and Mwandari Health Centres in the Bole District of the Savannah Region of Ghana as the comparison sites. The control district was selected purposively based on certain similar characteristics it shares with the intervention district. The two districts were one until 2004 when the Sawla-Tuna-Kalba district was carved out of the Bole district. Thus, they are twin districts. Both districts share boundaries with La’cote Dvore, they are both cosmopolitan districts with the inhabitants in both made up of Gonjas, Brifors, Vaglas, Dagaabas, Mos, and Lobis. The main economy within these two districts is farming. The settlements and culture of these two districts are similar.

Sawla-Tuna-Kalba district is one of the seven districts in the Savannah Region of Ghana. It has a total population of 125,525 (projections from the 2010 Population and Housing Census) with 278 communities. It shares boundaries to South, with Bole District, to the North, with Wa West District in the Upper West Region, West Gonja District to the east, and to the west La cote d’Ivoire and Burkina Faso. The district has 22 health facilities with one polyclinic, seven Health Centres, and 14 CHPS facilities. Two of these facilities are privately owned with three under the Christian Health Association of Ghana (CHAG) and the rest under the Ghana Health Service.

The Bole district on the other hand is in the western corridor of the Savannah region of Ghana. It has a population of 77,422 (projections from the 2010 Population and Housing Census) with 187 communities. It shares borders with La cote d’Ivoire to the West, Tain district to the south, West Gonja Municipal and Central Gonja District to the east, and Sawla-Tuna-Kalba District to the North. The major towns in the district are Bole, Bamboi, Mwandari, Mankuma, Jama, and Tinga with Bole as the district capital. The district has one District Hospital, seven Health Centres (one owned by CHAG), and eighteen CHPS facilities.

The control health facilities within the districts were purposively selected based on certain features they share with the intervention health facilities. Amongst them is the cadre of staff such as Physician assistants, Midwives, community health nurses, general nurses, etc. Also, the locations of the facilities were taken into consideration, e.g. Kalba health center in the intervention district share boundary with La cote d'Ivoire likewise Mwandari Health Centre in the comparison district. Consideration was also given to the level of prescription of the facility, that is, all the health facilities (intervention and control) were at the same level of standard classification of the health system to minimize selection bias.

### Outcome measures

The primary outcome measures in this study are follows: number of ANC attendance, facility delivery (coded yes or no), number of postnatal care attendance and continuum of care. The continuum of care was defined as the percentage of mothers who attended ANC (8 or more), had facility-based delivery, and attended post-natal care within the first 24 hours post-delivery.

### Intervention

Our primary exposure of interest is the T4MCH intervention. Technology for maternal and child health (T4MCH) is an intervention introduced to assist in increasing MCH services utilization in some health facilities across Ghana via the delivery of SMS/voice messages to pregnant women. The intervention is novel with the first SMS/Voice message generated and sent to the pregnant women in the 4 health facilities on 1st August 2017.

This intervention was funded and implemented by the Savana Signatures. Savana Signatures is an NGO based in Tamale in the Northern Region of Ghana. They are funded by Global Affairs Canada and the lead partner, Salsan, which has put together a consortium that combines the successful features of T4MCH, with innovations from Mustimuhw Information Solutions.

The intervention works via a designed mobile application known as SGS collect. The app enables health workers to collect personal information of pregnant women attending antenatal services via a smartphone onto a platform called Kpododo. In addition, the app delivers weekly SMS to literate women in English and voice messages for non-literate women in their preferred language to constantly educate and remind them of MCH services at least twice a week. The content of the message includes the need for attending MCH services, the specific date and time to visit health facilities, and general education on self-care and pregnancy risk aversion.

It also has the Knowledge Sharing Sessions (KSS) component where health workers undertake health education on Maternal and Child Health services both at the community level and the facility using audio-visual aids such as laptop computers and projectors as well as a public address system. This also provides a platform for mothers to share ideas with one another on MCH issues.

Health workers within the project area received ICT tools such as mobile phones, laptop computers, projectors, and public address systems. They also received training on the effective use of ICT tools to design, package, and effectively communicate maternal health content to women.

The project was piloted in 2011 and went into its first implementation phase from 2012 to 2014 in larger hospitals in Tamale and some districts in the Northern region. In 2015, it was scaled up to 5 additional smaller facilities in the Northern region, and by 2016 the project beneficiaries had increased to 33 health facilities across the three northern regions of Ghana.

### Target population

The intervention targeted all pregnant women who had their first ANC visit in the intervention facilities. Thus, the focus of this study is to evaluate the impact of the intervention on women who did their first ANC visits at the facilities located within the intervention communities.

### Covariates

To assess the impact of the intervention on the primary outcome measures, the following variables were adjusted for in all the multivariable regression analyses: age in years, marital status, educational level of mothers, occupation, parity, gestation at registration, and distance to the health facility.

### Sampling design

All ANC registrants' records available for the first 2 months (August 1, 2017, to September 30, 2017) of implementation of the intervention in all the four implementing facilities were reviewed retrospectively from antenatal through to delivery and postnatal. Four other facilities similar to the intervention facilities were purposively sampled in the comparison district. All records of ANC registrants within the same period were reviewed retrospectively from antenatal through delivery to postnatal. In all, 530 ANC registrant’s records were reviewed out of which 469 with complete data (263 study group and 206 comparison group) were used for the study. Data extraction was done using a well-designed data extraction tool. This tool was divided into three parts: demographic history, health facility characteristics, and health facility utilization. The demographic characteristics include age, level of education, marital status, parity, and occupation. The health facility characteristics included proximity to health facility and staffing. On the other hand, the health facility utilization consisted of the number of antenatal care attendance, institutional delivery, and the number of postnatal visits. Data were extracted from antenatal, delivery, postnatal registers, and ANC records booklet of clients with the help of trained research assistants. This study was given a formal waiver for the need for consent by the Ghana Health Service Ethics Review Committee of the Research and Development Division.

### Statistical analysis

The data was entered and cleaned via MS Excel and exported to Stata MP version 17 (StataCorp LP, Texas, USA) for analysis. Pearson’s Chi-square and Fisher’s exact test statistics were used to assess the association between antenatal visits, place of delivery, postnatal visits, socio-demographic characteristics, and the intervention. $T$-test statistic was also used to assess the mean age at ANC registration between the intervention and the comparison groups. The study estimated average treatment effects (ATEs) of the intervention for the number of antenatal care attendance, skilled delivery, and postnatal services utilization using the doubly robust method (augmented inverse-probability weights (AIPW), and via matching on the propensity score). The choice of these doubly robust impact evaluation techniques emanated from the study design. This is not a before and after study because no baseline data was collected prior to the implementation of the intervention. Because of this limitation, we employed a more rigorous impact evaluation statistical methods such as matching techniques and inverse probability weighting methods that could be used to quantify the impact of health interventions in the absence of baseline data.

In the ATE or the intention to treat (ITT) estimate, all women registering at the intervention facilities were treated as having received the T4MCH intervention, regardless of whether they signed up to receive the intervention or not, that is, we defined as $ITT=\mathrm{Average}[\mathrm{Y}(\mathrm{i},1)-\mathrm{Y}(\mathrm{i},0)]$ where $\mathrm{Y}(\mathrm{i},1)$ is the estimate of the outcome measure for beneficiary $i$ who visited the intervention facilities and $\mathrm{Y}(\mathrm{i},0)$ is the counterfactual scenario. We did not conduct per-protocol analysis (average treatment effect on the treated) as our primary objective was to determine the average impact of a program on the population targeted by the program.

**Figure 1:**
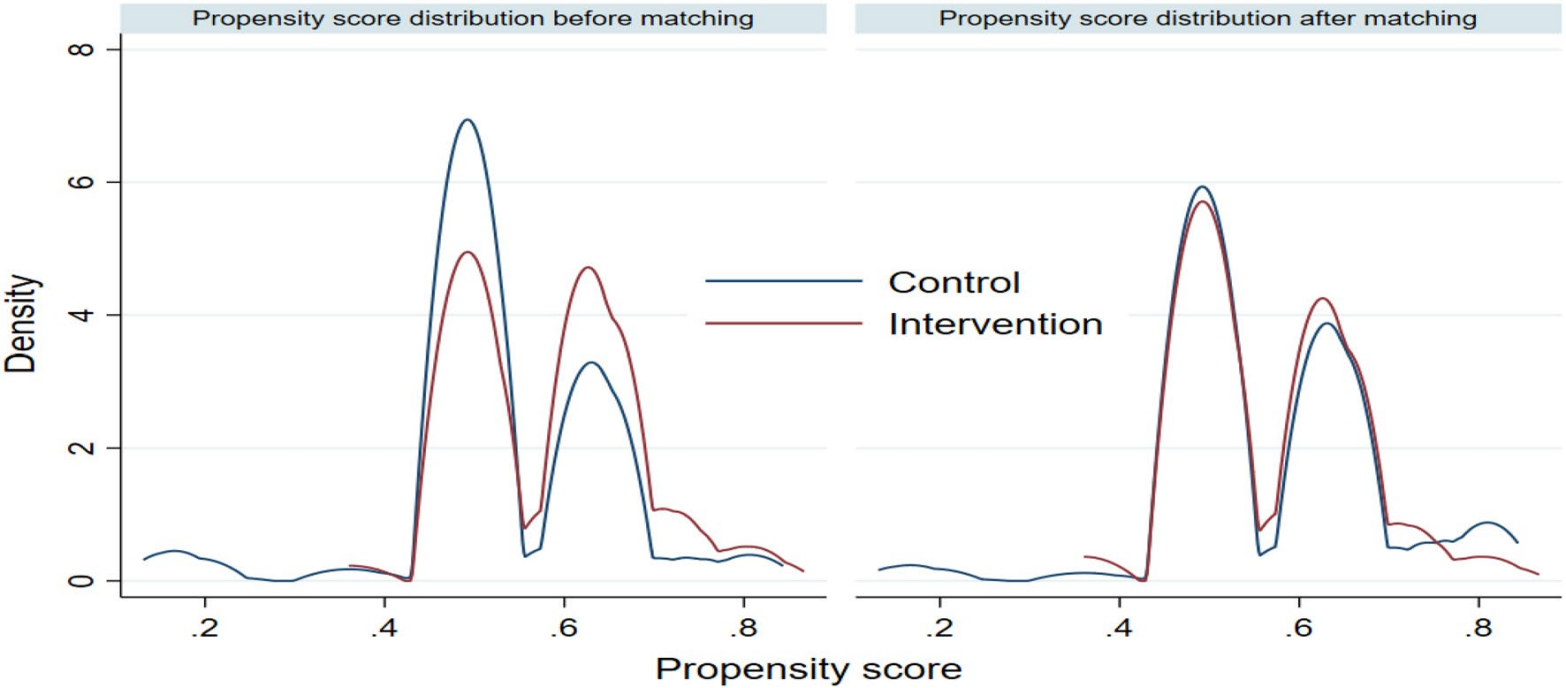
Kenel density of the propensity scores before and after matching for intervention and control populations.

We conducted balancing diagnostics to determine whether the matching improved covariate similarity index between the intervention and controlled study participants using a kernel density plot that compares the distribution of the propensity scores before and after matching. Since different impact estimation techniques may result in different impact estimates, we conducted a sensitivity analysis to show or prove that even when a different estimation procedure is used, we will still realize a positive impact or otherwise of the proposed intervention. The following sensitivity analyses were conducted: inverse-probability-weighted regression adjustment (IPWRA), 1:1, 1:2, 1:3 nearest neighbour matching, and propensity score matching. We modeled the number of ANC and PNC using the Poisson regression model with robust standard error to account for clustering at the facility level. Since the place of delivery is a binary indicator outcome measure, the logistic regression model was used. The baseline characteristics of program beneficiaries often differ systematically from those of non-beneficiaries, therefore, we accounted for systematic differences in baseline characteristics between T4MCH intervention beneficiaries and non-beneficiaries when estimating the effect of the intervention on outcomes of interest. We adopted matching procedures to reduce the covariate imbalance between the intervention and control groups since our data originate from an observational (or nonrandomized) study design (Fig. 1).

This study emphasizes the use of the size of the impact estimates and not the p values of the estimates based on the recommendation from the American Statistical Association which states that scientific conclusions and business or policy decisions should not be based only on whether a p value or statistical significance passes a specific threshold and a p value, or statistical significance, does not measure the size of an effect or the importance of a result and a p value does not provide a good measure of evidence regarding a model or hypothesis^7^.

### Ethical approval

Ethical clearance to conduct the study was given by the Ghana Health Service Ethics Review Committee of the Research and Development Division before the data collection. The approval reference is GHS-ERC 036/03/19. Permission was also granted by the District Directors of Health Services and Health Facility In-charges of the respective districts where the data was collected. The data reviewed were used solely for this research. We state that informed consent was not applicable in this study as we only analyzed secondary data from patients records that were de-identified. The Ghana Health Service Ethics Review Committee approved the study after carefully examining all the protocols. Permission was as well sought from the Sawla-Tuna-Kalba and Bole district health directorates.

## Results

### Socio-demographic characteristics of the study participants

A total of 469 health records (263 from intervention districts and 206 comparison districts) were reviewed retrospectively from the antenatal and postnatal registers. Table 1 illustrates the socio-demographic characteristics of pregnant women whose medical records were reviewed in the study. The results showed that almost half (49%) of the participants were between the ages of 20–29 years. There was a marginal difference between the mean age of those in the comparison and that of the study (26.2 ± 6.9 vs. 25.7 ± 6.9). Almost all the participants (98.1%) in both comparison and study facilities were married. The study also revealed that more than half (54.4%) of the participants live within a 0–5 km radius. In all, age, marital status and distance were significantly associated with program participation (p < 0.05; Table 1).

**Table 1:** Sociodemographic characteristics of the intervention and comparison groups.

Characteristics	Total (N = 469)	Control (n = 206)	Intervention (n = 263)	Chi-square estimate	p value
Age in years				9.698	0.021*
10–19	86 (18.3)	39 (18.9)	47 (17.9)		
20–29	230 (49.0)	113 (54.9)	117 (44.5)		
30–34	76 (16.2)	22 (10.7)	54 (20.5)		
35+	77 (16.4)	32 (15.5)	45 (17.1)		
Mean age ± SD	26.2 ± 6.9	25.7 ± 6.9	26.6 ± 6.9		0.141
Marital status				7.121	0.008*
Married	450 (96.0)	192 (93.2)	258 (98.1)		
Not married	19 (4.0)	14 (6.8)	5 (1.9)		
Educational level					0.850
Basic level	20 (4.3)	7 (3.4)	13 (4.9)		
Senior high school	11 (2.4)	5 (2.4)	6 (6.3)		
Tertiary	3 (0.6)	1 (0.5)	2 (0.8)		
No education	435 (92.8)	193 (93.7)	242 (92.0)		
Occupation					0.932
Formal	3 (0.60)	1 (0.5)	2 (0.8)		
Informal	441 (94.0)	195 (94.7)	246 (93.5)		
No occupation	25 (5.3)	10 (4.9)	15 (5.7)		
Parity				5.838	0.120
0	135 (28.8)	63 (30.6)	72 (27.4)		
1–2	160 (34.1)	76 (36.9)	84 (31.9)		
3–4	109 (23.2)	47 (22.8)	62 (23.6)		
5+	65 (13.9)	20 (9.7)	45 (17.1)		
Gestation at registration				0.477	0.788
1st trimester	240 (51.2)	107 (51.9)	133 (50.6)		
2nd trimester	169 (36.0)	71 (34.5)	98 (37.3)		
3rd trimester	60 (12.8)	28 (13.6)	32 (12.2)		
Distance(km) to facility				10.676	0.014*
0–5	263 (56.1)	120 (58.3)	143 (54.4)		
6–10	95 (20.3)	36 (17.5)	59 (22.4)		
11–20	70 (14.9)	24 (11.7)	46 (17.5)		
21+	41 (8.7)	26 (12.6)	15 (5.7)		

p value notation: ***p < 0.001, **p < 0.01, *p < 0.05.

### Effects of T4MCH on ANC attendance, skilled delivery, PNC utilization, and continuum of care

The T4MCH intervention increased antenatal care attendance, facility delivery, postnatal care and continuum of care by 18 percentage points (ppts) [95% CI − 17.0, 52.0], 14 ppts [95% CI 6.0%; 21.0%], 27 ppts [95% CI 15.0, 26.0] and 15.0 ppts [95% CI 8.0, 23.0] respectively. Table 2 shows the detailed results of the different impact estimation techniques used in quantifying the impact of T4MCH intervention on maternal and child health service utilization.

**Table 2:** Impact of T4MCH intervention on antenatal care attendance, skilled delivery, postnatal care attendance and continuum of care in the Sawla-Tuna-Kalba districts.

Impact estimation methods	Antenatal care attendance	Facility delivery	Postnatal care attendance	Continuum of care
	ATE [95% CI]	ATE [95% CI]	ATE [95% CI]	ATE [95% CI]
Augmented inverse probability weighted regression adjustment based on propensity scores	0.18 [− 0.17, 0.52]	0.14 [0.06, 0.21]***	0.27 [0.15, 0.26]***	0.15 [0.08, 0.23]***
Sensitivity analysis				
Inverse-probability weighted regression adjustment based on propensity scores	0.16 [− 0.18, 0.52]	0.14 [0.06, 0.21]***	0.27 [0.18, 0.38]***	0.14 [0.07, 0.22]***
Propensity score matching	0.24 [− 0.12, 0.59]	0.16 [0.08, 0.23]***	0.28 [0.18, 0.38]***	0.16 [0.09, 0.24]***
1:1 nearest neighbour matching	0.21 [− 0.14, 0.56]	0.14 [0.07, 0.22]***	0.27 [0.17, 0.38]***	0.14 [0.07, 0.22]***
1:2 nearest neighbour matching	0.20 [− 0.14, 0.55]	0.14 [0.07, 0.22]***	0.27 [0.17, 0.37]***	0.15 [0.07, 0.22]***

ATE is the average treatment effect, p value notation: ***p < 0.001, **p < 0.01, *p < 0.05. We converted the incident rate estimate from Modified Poisson model to percentage point change. That is, $Percent Point Change=(IR-1)\times 100\%$ where IR is the estimated incident rate of antenatal care attendance and postnatal care over the entire spectrum of the study.

## Discussion

Technology for maternal and child health intervention is implemented with the primary objective of improving maternal and child health services utilization. The study found that T4MCH intervention improved antenatal care attendance, skilled delivery, PNC services utilization, and continuum of care. Although the intervention had no statistically significant effect on ANC services utilization at the intervention district compared to the controlled districts, there was still a clinically significant increase in ANC attendance of about 18 percentage points. Continuum of care that involves the same mother attending ANC, delivering in a recommended health facility and attending postnatal care improved after the implementation of the intervention. We emphasized that an improvement in ANC attendance, facility-based delivery, and PNC services utilization and overall continuum of care will translate into a reduction in the risk of maternal and neonatal mortality as well as morbidity.

The intervention had a positive impact on skilled delivery via educating women on MCH services and reminding them of their due dates for delivery. This finding is consistent with similar studies in Ethiopia^5^, Zanzibar^8^, Nigeria^9^, India^10^ and Kenya^11^. These studies indicated that mobile messaging to educate and remind women on the need for facility delivery significantly increased skilled delivery at their respective study sites compared to comparison sites. Thus, this intervention could increase facility delivery when applied in similar settings.

We are cognizance of the fact that other factors besides the proposed mobile messaging intervention in this study may contribute to facility delivery as findings from other studies in Nigeria^12^, India^13^, Bangladesh^14^, and Rwanda^15^ did not show a significant impact of similar interventions. The variations could be due to geographic and cultural differences in the study settings.

The intervention increased PNC attendance because of the prompt reminder of mothers on PNC visits as well as educating them on the importance of PNC services may have motivated them to utilize PNC services at the study site. This is consistent with similar studies in Ethiopia^16^, Nigeria^17,18^, Vodopivec-Jamsek^19^, and Koshy^20^ indicated that sending reminder messages to mothers for PNC attendance significantly improved the rate at which these mothers attended scheduled PNC. Thus, this intervention could increase postnatal and other services when replicated in similar settings. In contrast, a study in Rwanda^15^ found that mobile messaging interventions had no significant effect on postnatal care attendance.

Other related studies in Guatemala^15^, and Bangladesh^21^ showed that mobile messaging did not influence the utilization of their respective services. The conflicting findings of the impact of the intervention may be attributed to the implementation strategy adopted in the different countries. The prompt reminder sent to pregnant women to attend scheduled ANC visits increased the number of ANC visits in the intervention district compared to the control districts. This finding is consistent with similar studies conducted in Ethiopia^22^, Zanzibar^23^, and Kenya^11^. The analysis of the findings from previous studies and our current study indicate that text and audio message-based interventions may improve care-seeking behavior among mothers if well implemented.

## Limitations

Several unknown factors including other interventions might have contributed to the observed change in health service utilization among women of reproductive age. Since the data was not obtained via experimental design, there may be systematic differences in the unobserved factors that could potentially affect our outcome measures of interest and mask the effect of our intervention. Although rigorous statistical methods were employed to quantify the impact of the intervention on these outcome measures, caution must be applied when interpreting the results as the study may suffer from this unobserved bias including socio-cultural beliefs and inherent individual level risk aversion that could influence the outcome measures. For instance, ANC attendance may increase not because of the proposed intervention, but because there were some pregnancy complications identified early in pregnancy by a health professional may automatically increase the number of ANC appointments based on the encouragement received from her care providers.

Although matching was done based on observed covariates, this method of analysis may not be able to control for the unobserved factors that may affect both program participation and outcome measures differently in the intervention and the control districts and may bias the study. That notwithstanding, the application of more rigorous statistical methods for assessing the impact of health intervention using observational study and the series of sensitivity analyses conducted make the study findings reliable and fit for the purpose.

## Conclusions

The study found that T4MCH intervention in the Sawla-Tuna-Kalba district in the Savannah region impacted positively on maternal and child health services utilization in the district. T4MCH improved the overall continuum of care (antenatal care, facility delivery, and postnatal care). The intervention is recommended for a scale-up in other areas.

## Data Availability

The datasets generated and/or analyzed during the current study are not publicly available due to legal and ethical concerns but are available from the corresponding author on reasonable request.

## Abbreviations

ANC: Antenatal care
CHAG: Christian Health Association of Ghana
CHPS: Community Health-based Planning and Services
DHIMS2: District Health Information Management System
GDHS: Ghana Demographic Health Survey
GHS: Ghana Health Service
GSS: Ghana Statistical Service
ICT: Information Communication Technology
MCH: Maternal and Child Health
MOTECH: Mobile Technology for Community Health
NHIS: National Health Insurance Scheme
PNC: Postnatal Care
SGS: Savana Global Solutions
SMS: Short Message System
T4MCH: Technology for Maternal and Child Health
UNFPA: United Nations Fund for Population Activities
UNICEF: United Nations International Children Emergency Fund
USAID: United States Agency for International Development
WHO: World Health Organization
WIFA: Women In Fertility Age

## References

[1] WHO. Maternal mortality 16. 3–7 (2018).

[2] GSS/GHS/ICF International. *Ghana Demography and Health Survey* (2014). 10.1007/s13398-014-0173-7.2.

[3] WHO (2015). WHO|Applying the Lessons of Maternal Mortality Reduction to Global Emergency Health.

[4] Lefevre AE (2017). Mobile technology for community health in Ghana: What happens when technical functionality threatens the effectiveness of digital health programs?. BMC Med. Inform. Decis. Mak..

[5] Ghana Health Service. District Health Information Management System 2(DHIMS2) (2018).

[6] T4MCH. Technology for Maternal and Child Health (T4MCH) Annual Report-April to March, 2018 (2018).

[7] Greenland S (2019). Valid p-values behave exactly as they should: Some misleading criticisms of p-values and their resolution with s-values. Am. Stat..

[8] Lund S (2012). Mobile phones as a health communication tool to improve skilled attendance at delivery in Zanzibar: A cluster-randomised controlled trial. BJOG Int. J. Obstetr. Gynaecol..

[9] Ango U (2018). Effect of health education intervention on knowledge and utilization of health facility delivery services by pregnant women in Sokoto State, Nigeria. Int. J. Contemp. Med. Res..

[10] Ilozumba O (2018). The effect of a community health worker utilized mobile health application on maternal health knowledge and behavior: A quasi-experimental study. Front. Public Health.

[11] Fedha T (2014). Impact of mobile telephone on maternal health service care: A case of Njoro division. Open J. Prev. Med..

[12] Nj, U. *et al.* Journal of Community Medicine & Impact of Health Education on Knowledge and Access to Delivery Care Services by Women among Edu Local Government Area, Nigeria **7**.

[13] Prinja, S. *et al.* Impact of m-health application used by community health volunteers on improving utilisation of maternal, new-born and child health care services in a rural area of Uttar Pradesh **22**, 895–907 (2017). 10.1111/tmi.12895 28510997

[14] Alam, M., Este, C. D., Banwell, C. & Lokuge, K. The impact of mobile phone based messages on maternal and child healthcare behaviour : A retrospective cross-sectional survey in Bangladesh 1–12 (2017). 10.1186/s12913-017-2361-6. 10.1186/s12913-017-2361-6 PMC5482970 28645278

[15] Ruton H (2018). The impact of an mHealth monitoring system on health care utilization by mothers and children: An evaluation using routine health information in Rwanda. Health Policy Plan..

[16] Shiferaw S (2016). The effects of a locally developed mHealth intervention on delivery and postnatal care utilization; A prospective controlled evaluation among health centres in Ethiopia. PLoS One.

[17] Idowu A (2014). Role of reminder by text message in enhancing postnatal clinic attendance Vaginal delivery after placental abruption and intrauterine fetal death, including failed cases. Int. J. Gynecol. Obstet..

[18] Jibril UN (2017). Impact of health education intervention on knowledge and utilization of postnatal care services among women in edu local government of Kwara State, Nigeria. J. Basic Clin. Reprod. Sci..

[19] Vodopivec-Jamsek V, de Jongh T, Gurol-Urganci I, Atun RCJ (2012). Mobile phone messaging for preventive health care (Review). Cochrane Database Syst. Rev..

[20] Koshy E, Car J, Majeed A (2008). Effectiveness of mobile-phone short message service ( SMS ) reminders for ophthalmology outpatient appointments. Observ. Stud..

[21] Biswas KK, Health MBA, Hossain A, Chowdhury R, Statistics MS (2017). Using mHealth to support postabortion contraceptive use: Results from a feasibility study in Urban Bangladesh. Corresp. Author.

[22] Atnafu A, Otto K, Herbst CH (2017). The role of mHealth intervention on maternal and child health service delivery: Findings from a randomized controlled field trial in rural Ethiopia. mHealth.

[23] Lund, S. *et al.* Mobile phones improve antenatal care attendance in Zanzibar: A cluster randomized controlled trial. 1–10 (2014). 10.1186/1471-2393-14-29 PMC3898378 24438517

